# Progressive Reduction of Iconic Gestures Contributes to School-Aged Children’s Increased Word Production

**DOI:** 10.3389/fpsyg.2021.651725

**Published:** 2021-04-26

**Authors:** Ulrich J. Mertens, Katharina J. Rohlfing

**Affiliations:** Psycholinguistics, Faculty of Arts and Humanities, Paderborn University, Paderborn, Germany

**Keywords:** word learning, child language acquisition, iconic gestures, reduction, economic principle of communication

## Abstract

The economic principle of communication, according to which successful communication can be reached by least effort, has been studied for verbal communication. With respect to nonverbal behavior, it implies that forms of iconic gestures change over the course of communication and become reduced in the sense of less pronounced. These changes and their effects on learning are currently unexplored in relevant literature. Addressing this research gap, we conducted a word learning study to test the effects of changing gestures on children’s slow mapping. We applied a within-subject design and tested 51 children, aged 6.7 years (*SD* = 0.4), who learned unknown words from a story. The storyteller acted on the basis of two conditions: In one condition, in which half of the target words were presented, the story presentation was enhanced with progressively reduced iconic gestures (PRG); in the other condition, half of the target words were accompanied by fully executed iconic gestures (FEG). To ensure a reliable gesture presentation, children were exposed to a recorded person telling a story in both conditions. We tested the slow mapping effects on children’s productive and receptive word knowledge three minutes as well as two to three days after being presented the story. The results suggest that children’s production of the target words, but not their understanding thereof, was enhanced by PRG.

## Introduction

### Reduction in Spoken Language and Gestures

How people structure information in speech depends on various factors, including what is assumed to be known, what kind of information is considered important, and what information the speaker wishes to focus on (e.g., Arnold et al., 2013). In this vein, studies on speech have shown that speakers exclude information when they tell a story for the second time to the same interlocutor and that stories told for the second time contain fewer details and fewer words (Galati and Brennan, 2010). Moreover, when referring to the same entity repeatedly, a speaker reduces the full lexical form by replacing it with a pronoun or a zero anaphora (e.g., Fowler et al., 1997; Galati and Brennan, 2010). Another form of reduction occurs when a word is produced less intelligibly (Bard et al., 2000, p. 2) by shortening its vocalization duration (Jescheniak and Levelt, 1994; Griffin and Bock, 1998; Bell et al., 2009; Lam and Watson, 2010) and producing it without pitch accent (Gregory, 2002; Watson et al., 2008). Overall, these kinds of reductions occur during an interaction for predictable (Haspelmath, 2008) or already known referents. The advantage of using less information is a phenomenon already well studied and is related to the economic principle of communication (for an overview, see Arnold et al., 2013).

Similar to verbal behavior, gestures that encode the same referent vary in their quantitative and qualitative aspects to adapt to the listener’s communicational needs (Gerwing and Bavelas, 2004; Galati and Brennan, 2014; Bohn et al., 2019) and in the interaction progress that contributes to emerging common knowledge (Clark, 1996). The similarity between verbal and gestural behavior is reflected in the current literature assuming that gesture and speech use the same communication planning processes (McNeill, 1992; Kendon, 2004). The two modalities function as one integrated system and are manifested in its temporal alignment (e.g., Jesse and Johnson, 2012; Esteve-Gibert and Prieto, 2014), in similar semantics (McNeill, 1992; Kendon, 2004), and in pragmatic aspects (e.g., Kelly et al., 1999).

Based on the well-acknowledged view that gesture and speech form an integrated system, in our study, we reasoned that speakers’ gestures undergo similar changes as speech forms (Galati and Brennan, 2010, 2014). In this vein and focusing on iconic gestures, which are gestures that bear semantic information about objects and actions, Galati and Brennan (2014) showed that gestures become attenuated in size and iconic precision when produced for a known interlocutor compared to an unfamiliar interlocutor. Similar to lexical forms in Galati and Brennan (2010), shared knowledge was visible in gestures in the form of a reduction. Similarly, Gerwing and Bavelas (2005) revealed that with increased, mutually shared knowledge, gestures become physically more schematic while simultaneously becoming conceptually more complex. Whereas the dimensions of reduction are still largely unexplored (Koke, 2019), it seems that interlocutors with a certain degree of shared knowledge use less accurate gestures than interlocutors without shared knowledge (Gerwing and Bavelas, 2004). The latter type of interlocutors (without shared knowledge) displayed more elaborated, informative and precise gestures (Gerwing and Bavelas, 2004). Similarly, Jacobs and Garnham (2007) demonstrated an effect on adult participants’ gestures that pertains to the interlocutors’ established common ground: Gestures became less complex, precise, and informative when speakers communicated about toys with which listeners had also played. Along the same lines, Holler and Wilkin (2011) demonstrated that interlocutors, who talked about shapes on cards in order to sort them, mimicked each other’s gestures during the dialog and that, as their mutually shared understanding increased, their gestures were produced less precisely. Overall, a reduction of gesture movements during an interaction and the loss of particular semantic aspects could be observed. It should be noted, however, that the reduction did not cause a loss of information in the context of the conversation. Instead, the relevant semantic information within the reduced gestures was available for the listener at any time because the listener could rely on the interaction history to link reduced gestures to referents introduced earlier on (Holler and Wilkin, 2011; Hoetjes et al., 2015).

In sum, the reviewed literature suggests that gesture production is adaptive to the listener’s emerging knowledge. The body of research also supports cross-situational processing mechanism in memory: More specifically, an aggregation of features that seems to form an overreaching element that is used in a contextualization process. In this process, an ongoing event is interpreted in light of the emerging knowledge of the interlocutors. However, direct empirical evidence for the effects of adapted (i.e., reduced) gestures for learning is currently lacking.

### Learning With Iconic Gestures

In contrast to the advantage of adapted gestures, gestural behavior itself is largely demonstrated to support language learning (see, e.g., Rohlfing, 2019 for a recent review). Several studies report an improvement in word learning for preschool children (e.g., Vogt and Kauschke, 2017a), elementary school students (e.g., Nooijer et al., 2014), and adults (e.g., Goodrich and Hudson Kam, 2009) in a word learning scenario in which iconic gestures accompany target words. However, most existing studies focus on younger children, thus, the evidence for older children is scarce (Rohlfing, 2019).

In the literature, two explanations are provided for the effectiveness of learning with gestures with regard to younger children. First, iconic gestures semantically enrich the encoding of unknown words (Capone Singleton, 2012) thus contributing to a long-term learning effect (McGregor et al., 2009), also referred to as slow mapping effect (e.g., Munro et al., 2012). In other words, new information is first processed in working memory (fast mapping) and then stored in long-term memory (slow mapping). According to word learning studies, the transition from working to long-term memory involves cognitive processes during sleep (Wojcik, 2013). These consolidation processes yield a memory trace that supports the retention of a novel word (e.g., Munro et al., 2012) and become visible as consolidation effect (for an overview, see Dudai, 2004; Wojcik, 2013). As already mentioned, in word learning, the contribution of iconic gestures was related to deeper processing: When a learner sees gestures performed, they evoke semantic elements that are not yet part of the word’s mental representation (Kita et al., 2017). Consequently, binding a relation between the entity perceived (e.g., a practical action) and its abstracted features in the form of a gesture results in a richer internal representation that requires a deeper level of processing (Goldstone and Son, 2005; McNeil and Fyfe, 2012; Kita et al., 2017). In turn, a deeper level of processing seems to leave a greater memory trace (McGregor et al., 2009; Son et al., 2012). Other explanations for the beneficial effect of iconic gestures focus on gestures that are used by the learner. In these situations, the use of iconic gestures lightens the demands on the learner’s working memory (e.g., Baddeley, 1986; Goldin-Meadow et al., 2001; Ping and Goldin-Meadow, 2010; Cook et al., 2012). For example, Goldin-Meadow et al. (2001) showed that children recalled a list of words better when they were allowed to gesture than when they were not.

Summarizing the existing research, Rohlfing (2019) points to the evidence that gestures support the learning of various word classes: nouns, verbs, and prepositions. While the acquisition of various word classes benefit from iconic gestures, the GSA framework, which is based on the idea that gestures arise from underlying motor or visual imagery, suggests that verbs require “complexive” attributes (Nomikou et al., 2019, p. 9) that might be better reflected in a multimodal way. This suggestion is grounded in empirical evidence that shows, for example, that children gesture more when describing a verb compared to a noun (Hostetter and Alibali, 2008, 2019; Lavelli and Majorano, 2016). Studies that investigated children’s word acquisition support this finding by demonstrating that when children observe iconic gestures their verb learning benefits from this observation (e.g., Mumford and Kita, 2014; Aussems and Kita, 2020). Mumford and Kita (2014) argue that iconic gestures guide children’s attention towards particular features of a scene which can enhance their semantic representation of unfamiliar verbs. Aussems and Kita (2020) demonstrated that iconic gestures foster the learning of locomotion verbs by preschool children. Yet another study showed that primary school children benefit from iconic gestures when learning locomotion verbs, but no enhanced learning effect was observed for object manipulation and abstract verbs (Nooijer et al., 2014). This finding indicates that iconic gestures’ influence on verb learning varies between verb categories.

Both explanations—to enrich the encoding semantically and to lighten working memory—that regard the facilitative effect of iconic gestures on word learning account for the effect that a single gesture has during a learning experience. We now turn to the questions of how and in what manner multiple presentations of a gesture can enrich the encoding of words.

### Learning With Variations of Gestures

To our knowledge, variation in iconic gestures has not been considered in word learning studies to date. Although the phenomenon of reduced gestures seems natural, it has not been studied systematically during learning situations. When gestures were used to support word learning in previous studies, they remained unchanged even when presented several times. In these studies, when the gesture consistency was an issue, it was achieved by presenting participants with gestures of video-recorded persons (e.g., Vogt and Kauschke, 2017a) or programmed social robots (e.g., Vogt et al., 2017). In contrast to gesture consistency, few studies tackled the issue of gesture reduction. Variation in gestures can be achieved in manifold ways and can occur in all gesture phases: preparation of the gesture, in which the hand starts to move from a resting position, the stroke, when a peak in movement is performed, and the retraction phase, in which the hand(s) switch to a rest position or to another gesture (Kendon, 1972, 1980; see for overview: Wagner et al., 2014).

One possibility to vary a gesture is to provide different aspects that refer to a specific referent. This is particularly relevant for iconic gestures that convey semantic information through their form (as in McGregor et al., 2009). For example, showing how an object falls could be depicted in a reduced iconic gesture by a quick hand movement that uses a downward movement. This event could also be depicted with an even more reduced gesture using only one finger. In contrast, the full gesture could involve an arm movement to depict the length of the downward movement, while the hand would additionally depict semantic features of the object.

It has been observed that such a reduction occurs naturally when speakers repeatedly refer to the same referent. They usually reduce some properties of the gesture without changing or losing the core meaning of the gesture (Gerwing and Bavelas, 2004; Holler and Wilkin, 2009, 2011; Galati and Brennan, 2014; Hoetjes et al., 2015; Bohn et al., 2019). As already stated above, the reduction of gestural presentation is not only a byproduct of emerging common knowledge: When the form properties change, the semantic information of the gesture changes as well. In the following paragraphs, we present arguments for why progressively reduced gestures, rather than gestures that are presented in the same manner, might improve learning.

First, learners aggregate information across different experiences with a novel referent and its labeling to discover the relevant properties and features (Yu and Smith, 2007). Following evidence provided above suggesting that gestures contribute to the semantic encoding (McGregor et al., 2009; Capone Singleton, 2012; Vogt and Kauschke, 2017b), we assume that gestures are part of semantic knowledge that is generated during exposure and will be used for learning. We further reason that children’s semantic knowledge is even more enhanced when learners experience different versions of a gesture because different semantic features of the referent are embodied in each version. In addition, these semantic features become contextualized in the process of unfolding knowledge and might become conceptually more complex with each version (Gerwing and Bavelas, 2004). This contextualization process might require more cognitive effort from the learner to bind the different features in the sum as relevant for the referent. To put it in other words, each time the gesture is performed to supplement an unknown word, it will provide additional, relevant information that needs to be related to the word. This is because the gesture becomes more and more abstracted from the referent.

We argue that this contextualization, namely, to relate the abstracted (or reduced) content to the referent, is an effort that fosters a deeper memory trace. In a similar vein, Son et al. (2012) studied under what situational circumstances children generate relational information that leads to generalization across trials. They concluded that for a word to become generalized, there should not be too much concrete information involved in the labeling experience (Son et al., 2012, p. 9). When learning instances are too specific, this experience might activate only an immediate memory system and not generate any relational information. This work led us to hypothesize that the interpretation of several reduced features accumulated in gesture results in meaningful relationships between the depicted features and the concrete referent and, furthermore, contributes to children’s robust word learning.

Further support for our premise comes from research that shows that movements in the field of view have a distracting effect and can interfere with the participants’ task performance (Lavie, 1995, 2005; Rees et al., 1997; Forster and Lavie, 2008). Distractors that are unrelated to a task and only appear in the periphery attract participants’ attention when cognitive resources are available. More importantly, task-relevant distractors are just as likely to interfere with task performance as irrelevant distractors (Forster and Lavie, 2008). When applying Lavie’s attention theory to children who observe fully executed iconic gestures constantly, we derive the idea that these children pay attention to such gestures (Kelly et al., 2010; Wakefield et al., 2018a); however, seeing fully executed gestures multiple times might have a distracting effect (Forster and Lavie, 2008). Using our earlier example, a gesture that depicts the event of a falling object can be performed by raising the hand above the head and then moving the hand in a quick motion toward the floor or by a short movement with only one finger. As illustrated above, an interlocutor can gather the full meaning of a reduced gesture when it is performed in context. Paying attention to a fully executed gesture requires cognitive resources that are not directed to the accompanying word. This assumption is supported in studies showing that higher cognitive load is reflected in participants observing movements and solving linguistic tasks (Rees et al., 1997). In contrast, when observing a reduced gesture, a child might focus more on the accompanying word. As such, experiencing a reduced gesture depicting the event of falling down might distribute children’s attention more equally on the gesture as well as the word. Consequently, a rich memory of the referent can be created because cognitive resources are distributed more economically to build better-balanced relational structures between the semantic features in the gesture and the label.

In sum, our assumption that progressively reduced iconic gestures might foster a memory trace of an unknown word is based on the following: Their reduced movements (i) require a contextualization that let a relational structure between the word and the reduced features of the gestures emerge through aggregation of semantic features and (ii) are less distracting and can even create a processing focus on the label over time. The first premise pertains to cognitive learning mechanisms that appear to be activated during the observation of iconic gestures. For the second premise, we have argued that learning becomes enhanced due to more balanced distributed cognitive resources when observing progressively reduced iconic gestures. Together with the above-mentioned fact that reduction occurs in natural communication, these premises provide a basis for our study.

The main goal in our study was to investigate whether children are sensitive to reduced iconic gestures and whether their long-term word learning (production and reception) is enhanced when observing progressively reduced iconic gestures. Whereas the existing body of research focuses on preschool children (Rohlfing, 2019), we investigated older children to extend an existing body of research to which we can associate our study with respect to both advantages of (i) gestural presentation for unknown words as well as (ii) long-term memory. Studies have shown more potent effects for children when tested with delay to initial exposure to a target word (e.g., Munro et al., 2012). Furthermore, being aware that word learning comprises the acquisition of many word classes, our aim was to account for this diversity in our study design, for which we used nouns and verbs as target words.

## Materials and Methods

### Participants

Fifty-one first graders, including 25 females and 26 males from two schools in the region of Meerbusch (North-Rhine Westphalia) in Germany, participated in this study. The participants ranged in age from 6.0 to 7.4 years (*mean* = 6.7; *SD* = 0.4). Socioeconomic status data were not collected from children, but the population from which the sample was drawn was predominantly middle to upper-middle class.

### Stimuli

For our study, we used a word learning setting in which words are embedded within a story—a previously designed successful method for children (Nachtigäller et al., 2013; Vogt and Kauschke, 2017a). The story, target words, pictures, and iconic gestures were used from another word learning study (Vogt and Kauschke, 2017a,b). In their study, Vogt and Kauschke (2017a, b) demonstrated that preschoolers gained greater word knowledge when a speaker accompanied words with iconic gestures compared with attention gestures. For our study, we modified the story in terms of the frequency and the number of target words. In total, we embedded 4 nouns and 4 verbs within the story, and each of them occurred three times. Whereas the four nouns referred to animals, the verbs referred to locomotion. The eight words were German words chosen by Vogt and Kauschke (2017a) and identified as low-frequency words (University of Leipzig, 1998–2013). In support of this, four- and five-year-old German children (*n* = 16) were asked to name the stimuli, and none of the children could name any of the stimuli (Vogt and Kauschke, 2017a). We supplemented the ratings by asking adults (n = 10) to name the stimuli. Only one of the ten adults was able to name one word (a noun).

As in the original study, children watched a recorded person who told the story and accompanied the target words with gestures (Vogt and Kauschke, 2017a). Additionally, we extended the multimodal presentation of the target words by presenting reduced gesture versions (see Supplementary Material). To ensure consistent word pronunciation of the target words, we desynchronized gestures from the spoken word by performing the gestures shortly after the spoken word. This way, the stroke of the gesture was not synchronous with the target word. Instead, the gesture was presented right after the word was produced.

**FIGURE 1:**
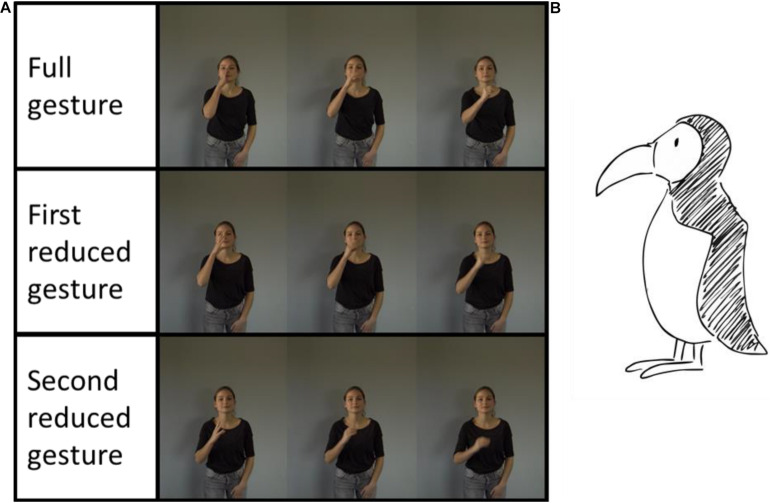
**(A)** The three gesture versions of the iconic gesture for the noun “auk”. The first row depicts the fully executed gesture; the second row depicts the first reduced gesture version; the third row depicts the second reduced gesture version. **(B)** The picture displays the referent “auk” (Copyright © 2013 Joy Katzmarzik leap4joy graphics; reprinted with permission).

We created two reduced gesture versions for each gesture. With every reduction, a gesture becomes less complex and less precise (Jacobs and Garnham, 2007) by lowering the gesture’s level of detail and shortening its trajectory (Galati and Brennan, 2014; Hoetjes et al., 2015). Reduced gestures for nouns and verbs were achieved by indicating the shape of an object and/or the action movement with less accurate spatial information about the referent’s location. For both word classes, this reduction led to a shortened duration of the gesture phases. In Figure 1, the fully executed gesture accurately depicts the shape and location of the auks’ beak, whereas the second reduced gesture only implies the beaks shape and its spatial location. Similar characteristics account for reduced gestures of locomotion verbs. For example, the fully executed gesture for “to creep” depicts the referent’s movements and a clear horizontal movement direction. The reduced gesture version indicates only a horizontal direction with the speaker performing an almost arcuate hand movement from the left to the right. For reduced gestures of both word classes, the stroke phase is not clearly separable from the preparation and retraction phases.

### Design and Procedure

For our investigation, we visited children at their respective schools for two sessions. We selected five different classes from two schools. Before starting the first session, the experimenter visited the children in their classroom to introduce himself and the project. The children’s parents were informed and asked for their consent by letter. The study commenced after parents provided written consent to their children’s participation, which is in accordance with Paderborn University’s ethics procedures for research with children. The procedure and consent forms were approved by the university’s ethical committee. The children also provided verbal consent before participating. Additionally, they were informed that they could discontinue the interaction at any time.

**FIGURE 2:**
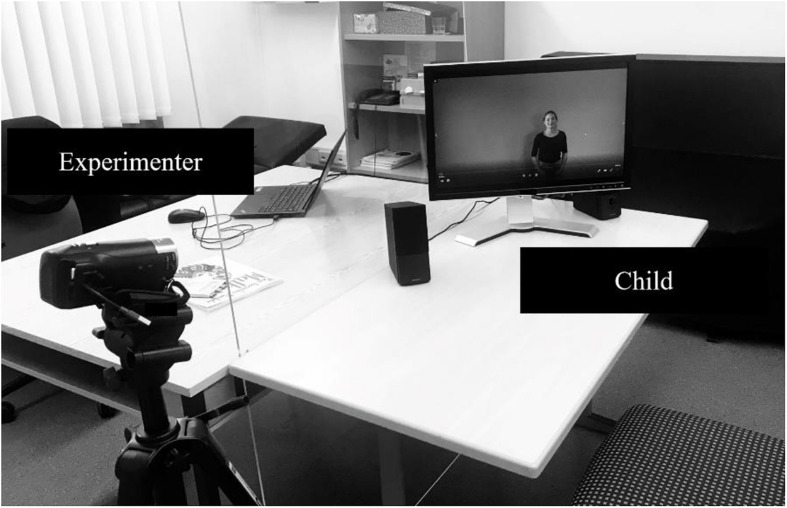
Study setup.

The two sessions for our investigation took place in a one-to-one constellation with only the child and the experimenter in the room. In both sessions, a child sat down in front of a monitor set up on a table. The experimenter was sitting at another table opposite the child. A plexiglass panel was placed between the tables as a precautionary measure due to the Coronavirus pandemic (see Figure 2). The first session lasted approximately fifteen minutes and the second session about five minutes. The children’s responses during the testing were videorecorded for later analysis. The experimenter was aware of the purposes and hypotheses of the study but blind to the gesture condition that a child experienced.

*Learning*: After a short chat about how the children feel being in first grade, the experimenter explained to the children that he wanted to show them a video of a young adult who would tell a story about her first-grade experience. After the child’s consent, the experimenter started the video.

**FIGURE 3:**
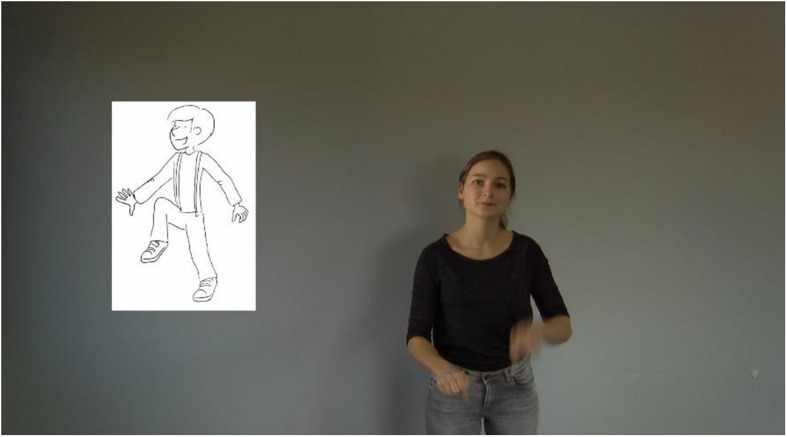
Recorded storyteller performing the gesture for the German target verb “*staksen”* [to lift the legs alternately]. Next to her, the referent appears as an image (Copyright © 2013 Joy Katzmarzik leap4joy graphics; reprinted with permission).

In the video, a woman told a story about animals and actions (that served as target words). We applied a within-subject design: To identify the effect of gesture reduction on children’s slow mapping of novel words, half of the iconic gestures became progressively reduced. For this, children were exposed to three versions of gestures that appeared progressively reduced. Furthermore, the story was designed for each target word to occur three times in succession, without other target words being mentioned. During this part, a picture with the referent was shown next to the speaker (see Figure 3). Showing children an image of a referent within the experimental setup is necessary for testing children’s word knowledge that was administered after the learning phase.

**FIGURE 4:**
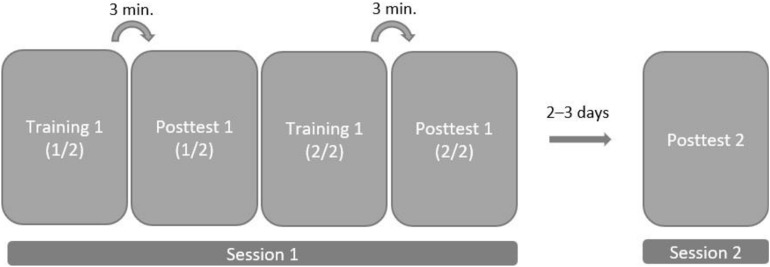
The study design: In the first session, the story was split into two parts. Each training part was followed by a posttest that was administered after a 3-minute delay. Posttest 2 took place during the second session.

In our pilot study, when we put eight target words that the children had to remember in one story, we obtained floor learning effects. Our interpretation was that recalling eight target words might have overwhelmed the children. Our attempts to reduce the load were successful, and we found that children performed better when they watched the story in two parts. For this reason, we first presented one part of the story (with four target words) and tested children’s learning performance after a break of three minutes. After testing children’s word knowledge, we continued with the second part of the story (with different four target words) that was followed three minutes later by a second test. According to this study design, children’s receptive and productive knowledge of the target words was assessed twice, once after each part (see Figure 4). This design raises the issue that children might be aware of the story’s purpose during the second part. Consequently, children might learn target words from the second part better. To avoid this bias, we created two story versions in which the target words were embedded differently. The target words that occur in the first part of the first story version were embedded in the second part of the second story version and the target words that occur in the second part of the first story version were embedded in the first part of the second story version. Every part contained four different target words (two nouns and two verbs). Furthermore, each story version was created in two ways, depending on whether target words were accompanied with progressively reduced iconic gestures (PRG), or fully executed iconic gestures (FEG). In total, we created four videos that differed in the order of the target words and the gesture versions (progressively reduced or fully executed) that accompanied the target word.

*Testing*: The main goal of this study was to identify the long-term effects of reduced gestures on children’s productive and receptive word knowledge. For this purpose, children’s slow mapping performance was assessed three minutes after hearing each part of the story. During the break between training and testing, the children were asked to color a picture. A further long-term effect on children’s receptive and productive knowledge was tested in the second session that took place two to three days after the first session. During the testing sessions, children were shown pictures of the target words, similar to those shown in the video. However, for the nouns, the pictures displayed the animals from a different perspective, and the verb pictures featured a girl instead of a boy. Throughout the testing, the experimenter provided neutral feedback to the children’s answers.

**FIGURE 5:**
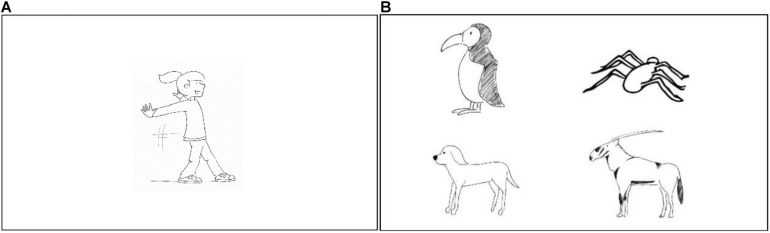
Monitor screen during **(A)** the production task and **(B)** the reception task. In the example for **(A)**, the girl is performing the action “to slide.” In the example for **(B)**, at the bottom right, the referent for the target word “beisa” is displayed. A distractor object that looks similar to the target referent is presented to the left of the “beisa”. In the first row on the left, another target word, “auk,” is presented as a distractor. Next to the “auk,” a random referent is shown as an additional distractor (Copyright © 2013 Joy Katzmarzik leap4joy graphics; reprinted with permission).

As mentioned above, our testing assessed children’s performance in word production and understanding. During the production task, the experimenter asked the child: “Can you tell me what kind of action the girl in the picture is performing?” or “Can you tell me what kind of animal is shown in the picture?” At the same time, the picture of the referent was shown on the monitor (see Figure 5). In the case when children did not provide an answer within five seconds after the question was raised, the experimenter asked the children if they had any idea. If another five seconds elapsed without an answer, the experimenter moved on to the next picture and said “no problem, let’s look at the next picture” or provided a similar form of reassurance. The experimenter also continued with the next picture when the children gave a correct or incorrect answer or made it explicit that they did not know the answer. In that case, the experimenter said, for example, “let’s look at the next picture.”

Children’s performance was scored according to a coding system. Children obtained (i) two points when both the onset and the offset of the word were correct and they used the correct number of syllables, (ii) one point when they produced either the onset or the offset of the word correctly and used the correct number of syllables, and (iii) zero points when they produced the word onset and the offset incorrectly or when the number of syllables was incorrect.

Fifteen percent of production responses were randomly selected and coded by an independent research assistant. We measured interrater reliability using Cohen’s Kappa (Cohen, 1960) and obtained an agreement of *k* = 0.92.

In the reception task, children were presented the target referent with three distractors; all referents formed a 2x2 arrangement (see Figure 5). The probability of choosing the correct answer by chance was 25%. The distractors in the arrangement included a picture similar to the target referent, another target picture out of our study (same word type), and a random picture. The testing started by asking the child, for example, “Can you touch the picture where you see the beisa?” When children did not point at the screen within five seconds of being asked the question, the experimenter asked again if they could point at the screen. If another five seconds elapsed without an answer, the experimenter moved on to the next referent and said to the child “It doesn’t matter, let’s look at the next picture” or provided a similar form of reassurance. The experimenter also continued to the next referent when the children pointed at the screen or made it explicit that they did not know the answer. The experimenter initiated this progression with words such as “let’s look at the next picture!” After testing session 2, each child’s performance was scored according to a coding system: Children obtained one point for each correct answer and zero points for an incorrect answer.

### Data Analysis

We applied an omnibus 3-way analysis with the independent variables gesture (progressively reduced iconic gestures (PRG), fully executed iconic gestures (FEG)) and time (T1, T2) for testing effects on nouns and verbs for both production and reception. Greenhouse–Geisser corrections were applied where necessary. Significant interaction effects were resolved by Bonferroni corrected *post hoc* pairwise comparisons. For the production task, we additionally conducted an item analysis. We first report on the production task before and then turning to the reception task.

## Results

### Word Production

Children’s performance was measured on a scale from 0 to 16 for word learning (8 points for words accompanied by progressively reduced gestures (PRG) and 8 points for words accompanied by fully executed gestures (FEG)). Children achieved a mean of 3.17 points (*SD* = 2.57; range: 0–10) during testing Session 1. During the testing Session 2 children achieved a mean of 3.80 points (*SD* = 2.58; range: 0–12). Their performance is displayed in Table 1.

**TABLE 1:** Children’s mean production scores (SD) in the testing Session 1 (T1) and testing Session 2 (T2).

	Production			
	T1		T2	
	PR	CF	PR	CF
Words	1.92 *(1.77)*	1.16 *(1.47)*	2.59 *(1.91)*	1.20 *(1.48)*
Nouns	1.08 *(1.23)*	0.75 *(1.26)*	1.24 *(1.35)*	0.67 *(1.10)*
Verbs	0.84 *(1.22)*	0.41 *(.75)*	1.35 *(1.39)*	0.33 *(1.33)*

*The maximum word score was 8 points with 4 points for nouns and verbs each. PRG = progressively reduced iconic gestures; FEG = full executed iconic gestures.*

**FIGURE 6:**
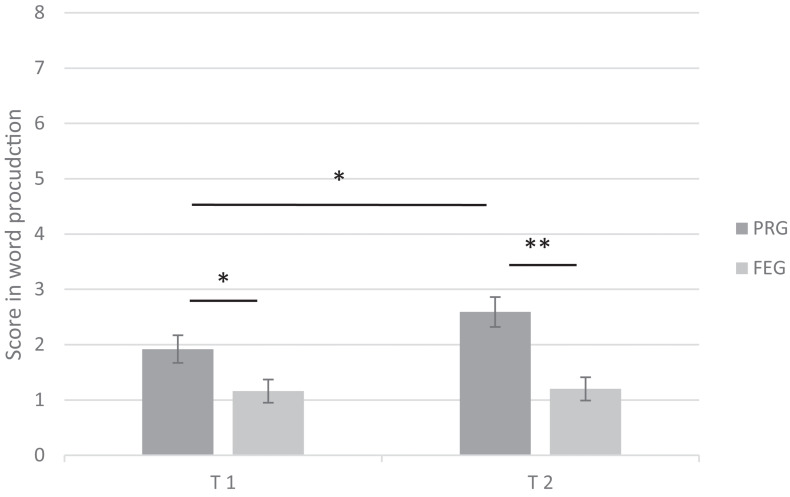
Children’s word production score (*SE*) at the first (T1) and second (T2) testing. PRG = progressively reduced iconic gestures; FEG = fully executed iconic gestures; children could score 8 points in both conditions, * *p* < 0.05, ** *p* < 0.01, *** *p* < 0.001.

The ANOVA confirmed an intermediate significant interaction effect gesture × time (*F*(1,50) = 5.55, *p* < 0.05, η*p*^2^ = 0.10), reflecting that children scored differently in the gestural conditions and that the difference between conditions depended on the time of retention. In *post hoc* analyses, multiple pairwise comparisons revealed that children achieved higher scores in Session 1 when words were presented with PRG than when words were accompanied with FEG (*p* < 0.05). Similarly, in Session 2, children scored higher in the PRG than in the FEG condition (*p* < 0.01). These results suggest that children’s word production was enhanced when they were exposed to presentation with PRG. Further analyses revealed that in the PRG condition, children achieved a higher score during T2 than during T1 (*p* < 0.05) suggesting that the effect of PRG became more pronounced over time (see Figure 6). For FEG, the *post hoc* analysis revealed no differences between T2 and T1 (*p* = 0.86). The ANOVA yielded no further significant interaction effect for gesture × word class × time (*F*(1,50) = 1.56, *p* = 0.22, η*p*^2^ = 0.03), word class × time (*F*(1,50) = 0.67, *p* = 0.42, ηp^2^ = 0.01) or gesture × word class (*F*(1,50) = 1.05, *p* = 0.31, η*p*^2^ = 0.02) indicating that nouns and verbs were produced similarly at both points in time and under both gesture conditions.

In the next step, we applied an item analysis to assess the item’s quality within the FEG and the PRG condition. The item difficulty ranges from 0.06 to 0.38 indicating that producing the target words can be considered as quite difficult for the participating children. Table 2 shows that the frequency distribution of item difficulty is lower for seven out of eight items within the PRG condition. The item “fennec”, however, was an exception, because it similarly difficult in both conditions. This analysis confirms that most items were learned more easily within the PRG conditions.

**TABLE 2:** Mean score, standard deviation (*SD*), and difficulty for each item (target word) in the PRG and FEG condition (PRG = progressively reduced iconic gestures; FEG = fully executed iconic gestures).

Item	Ralle		Alk		Fennek		Beisa	
	*“rail”*		*“auk”*		*“fennec”*		*“beisa”*	
Condition	FEG	PRG	FEG	PRG	FEG	PRG	FEG	PRG
Mean (*SD*)	0.32 (0.*73*)	0.76 (0.*95*)	0.16 (.*54*)	0.48 (0.*80*)	0.40 (0.*78*)	0.44 (0.*81*)	0.41 (0.*81*)	0.85 (0.*91*)
Item Difficultiy	0.16	0.38	0.08	0.24	0.20	0.22	0.21	0.42

**Item**			**Staksen**				**Retschen**	
	**Gliddern**		***“to lift the legs***		**Krauchen**		***“to slide***	
	***“to slide“***		***alternately“***		***“to creep“***		***backwards“***	
**Condition**	**FEG**	**PRG**	**FEG**	**PRG**	**FEG**	**PRG**	**FEG**	**PRG**

Mean (*SD*)	0.42 (0.*83*)	0.64 (0.*92*)	0.26 (0.*62*)	0.62 (0.*89*)	0.13 (0.*38*)	0.37 (0.*71*)	0.22 (0.*60*)	0.48 (0.*81*)
Item Difficultiy	0.21	0.32	0.13	0.30	0.06	0.18	0.11	0.24

### Reception Task

Children could score 8 points in the reception task (4 points for words accompanied by PRG and 4 points for words accompanied by FEG). In testing Session 1, children obtained a mean of 6.41 points (*SD* = 2.00; range: 0–8 points). During the testing in Session 2, children achieved a mean of 6.70 points (*SD* = 1.84; range: 2–8 points). Differentiating between word types (nouns and verbs), children could achieve 4 points for each word type (2 points for words accompanied by PRG and 2 points for words accompanied by FEG). For nouns, children obtained a mean of 3.08 points (*SD* = 0.73 ranging from 0–4) in testing Session 1. During testing in Session 2, children achieved a mean of 3.32 points (*SD* = 1.27 ranging from 0–4 points). With respect to verb reception, children obtained a mean of 3.36 points (*SD* = 0.65 ranging from 0–4) in testing Session 1. During testing Session 2, children achieved a mean of 3.42 points (*SD* = 0.99 ranging from 1–4). The probability to choose the correct answer by chance was at 25% within the reception task. With children’s responses being at 80% in testing Session 1 and 83% in testing Session 2 for words in general but also 77 % in testing Session 1 and 83% in testing Session 2 for nouns and 84% in testing Session 1 and 86% in testing Session 2 for verbs in specific, we can state that children performance in word reception was well beyond the chance level.

The ANOVA revealed no significant interactions, gesture × word class × time (*F*(1,50) = 1.73, *p* = 0.20, η*p*^2^ = 0.03), word class × time (*F*(1,50) = 0.93, *p* = 0.34, η*p*^2^ = 0.02), gesture × word class (*F*(1,50) < 0.01, *p* = 0.93, η*p*^2^ < 0.01), gesture × time (*F*(1,50) = 0.01, *p* = 0.92, η*p*^2^ < 0.01), revealing that the children’s performance in the reception task seems robust against the gesture presentation and time condition for nouns as well as verbs (see Table 3).

**TABLE 3:** Children’s mean reception scores (SD) in testing Session 1 (T1) and testing Session 2 (T2).

	Reception			
	T1		T2	
	PRG	FEG	PRG	FEG
Words	3.31 *(0.99)*	3.10 *(1.01)*	3.41 *(0.78)*	3.29 *(1.06)*
Nouns	1.55 *(0.64)*	1.53 *(0.09)*	1.73 *(0.57)*	1.59 *(0.70)*
Verbs	1.75 *(0.05)*	1.61 *(0.60)*	1.73 *(0.45)*	1.69 *(0.54)*

*The maximum of word score is 4 points with 2 points for nouns and verbs each. PRG = progressively reduced iconic gestures; FEG = fully executed iconic gestures.*

## Discussion

Whereas the economic principle of communication is well studied for verbal communication, little is known about means and effects of economic communication in gestural behavior. Aiming to close this gap, our study was designed to experimentally investigate the effects of progressively reduced iconic gestures (PRG) on children’s word learning at a mean age of 6.7 years (*SD* = .4). More specifically, we asked whether children’s slow mapping can be enhanced by presenting PRG in contrast to consistently fully executed iconic gestures (FEG). This new form of gestural presentation was motivated by two research strands: One strand includes studies demonstrating that iconic gestures comprise reductions of the referent’s semantic features (e.g., Kita et al., 2017). Along these lines, we reasoned that this reduction leads to a more abstracted presentation of the referent, which is important to induce deeper memory processing resulting in a better learning outcome (Mumford and Kita, 2014; Son et al., 2012). Additionally, our study was motivated by the finding that common ground between interlocutors affects their gesture performance in a way that their gestures become reduced, but the reduction causes no loss of information in the context of the conversation (e.g., Holler and Wilkin, 2011; Galati and Brennan, 2014; Hoetjes et al., 2015). We reasoned that repeatedly observing FEG can lead to distracting effects, whereas through PRG, cognitive resources are distributed more economically and thus better balanced for processing meaningful input from a gesture and its accompanied label (Forster and Lavie, 2008; Kelly et al., 2010). Combining these two research strands, we expected children to retain target words accompanied by PRG better than words accompanied by FEG.

In our study, children were presented with eight unknown words: four nouns and four verbs. The unknown words were embedded in a story. Applying a within-subject design, children received four target words presented by PRG and four other target words presented by FEG. All children participated in both conditions. Our analysis focused primarily on long-term effects because retaining a word for several minutes or several days indicates that the word has been acquired robustly (e.g., Munro et al., 2012; Wojcik, 2013). For this reason, children’s performance in word reception and production were assessed at two different points in time: after a delay of three minutes and after two to three days.

For word reception, we found no significant effect, neither when looking at the differences between the presentations nor when looking at what point in time the assessments occurred. We can therefore conclude that the reception of unknown words seems robust to our experimental manipulation. Furthermore and because of the high scores obtained in both conditions, our results suggest that first graders are generally strong in word reception. The referent’s picture might have been a beneficial (nonverbal) resource for formulating the correct answer. Thus, it seems reasonable that older children are experienced enough to recall a word meaning with the presentation of a picture’s referent—even if it is displayed from a different perspective. In contrast to our results, strong long-term effects on word reception were reported for younger children at the age of two, when the learning process was supported by iconic gestures (Horst and Samuelson, 2008; McGregor et al., 2009; Munro et al., 2012). It seems likely that the word reception test in our study was too easy for the children, which is a limitation of our design. In future studies, it would be more appropriate to design a testing procedure that requires the reception to be embedded in more demanding tasks, such as the understanding of text that contains the target words.

Regarding word production, we found that children were able to learn target words accompanied by PRG more successfully than words accompanied by FEG. In accordance with previous studies that revealed long-term effects of learning with gestures (McGregor et al., 2009; Munro et al., 2012), we found that the advantage of the PRG presentation was more pronounced when tested two to three days after initial exposure. We explain this as being a result of children’s greater sensitivity to a word’s presentations accompanied by PRG because the children experienced various forms of the gesture that might have fostered rich word concepts. These concepts were then available for the children during the assessment of their word production performance. The concept richness might be due to a greater variation in semantic properties in PRG, which are all related to each other. For example, the fully extended gesture for “to creep” contains several finger movements and a long horizontal trajectory, while the second reduced form contains no finger movements and only a short, almost arched trajectory. By removing semantic aspects from an iconic gesture, children might focus on the remaining semantic aspects from the reduced gesture. This way, children are exposed to a broader spectrum of semantic aspects within gestures that allows them to build a more substantial memory trace. In this form of gesture support, the variety of gestures includes a higher level of multimodal information. Thus, children can build up their semantic knowledge by continuously picking up semantic features that are novel or incongruent with their current word conception. This selected and contextualized exposure to various semantic features fosters the process of elaborating an existing representation and leads to a broader relational knowledge of the referent event. In support of this explanation, much research has emphasized that sematic knowledge drives the successful retrieval of a word’s label for production (e.g., McGregor et al., 2002; Capone and McGregor, 2005; Capone Singleton, 2012).

While variations in gesture lead to a more complete and distinct representation in memory, it should be noted that the presentation of PRG included consistency in the presentation of the target word. This way, in repetitions of the presentation, the word became the invariant element (Son et al., 2012). Consequently, the word likely became a focus leading to a stronger memory trace by serving as a strong link between the semantic features within the gesture versions and the label. We argue that this focus also accounts for the beneficial effect of the PRG presentation that leads to stronger word production performance in a long-term. Son et al. (2012) have demonstrated that when cognitive effort is intensified to interpret perceptual events in the context of a word, a stronger relation between the label and the referent is created. The cognitive effortful processes that include extracting, supplementing, and contextualizing semantic features from PRG is likely to provide the semantic link that is needed to retain and recall a word in the long-term (Capone Singleton, 2012).

Experiencing RPG can clearly be viewed as contextualization that is taking place with regard to the ongoing gain of knowledge that the child is experiencing. However, it is important to note that following this explanation, it might also be possible that children’s learning would benefit from presenting words with gestures that are not reduced but are instead presented each time differently. Further studies need to account for this alternative explanation. In line with our argumentation highlighting the relevance of semantic features in the facilitation process, we hypothesize that three unrelated gestures will not have the same beneficial effect on the production of unknown words.

As discussed above, our study demonstrates that children’s slow mapping was enhanced when they were exposed to PRG gestures. To identify if specific stimuli drive this finding, we compared how well children learned each word in the PRG and the FEG conditions. The analysis revealed that all words, except the noun “fennec”, were easier to produce when children observed PRG. Producing the word “fennec” appears to be equally challenging within the PRG and the FEG conditions. Interestingly, the gesture versions for “fennec” are executed with no movements within the stroke phase (the phase that contains the maximum semantic information density). All other gestures included movements within the stroke phase. We suggest that reducing a gesture that is void of movement in the stroke phase generates a lower variety of semantic features and can be interpreted effortlessly. The lower variety of semantic features, which seems to be easily processed, does not appear to contribute to the current internal word representation. The iconic gesture for “fennec” depicted the large ears of the animal. While the fully executed gesture version depicted the ears at an appropriate position on the head, the reduced gesture versions depicted the ears at less accurate positions. The reduced iconic gesture versions of other referents, like the peak of the auk, were reduced more strongly, involving a reduction of both the object (the peak) and the spatial position (see Figure 1). However, it also stands to reason that the item difficulty for “fennec” is similar in both conditions because it was simply not sufficiently reduced and not because of the missing movements within the stroke phase.

## Outlook

Our study indicates that PRG enhanced children’s long-term word production in general, but no differences in learning nouns versus verbs were found. These findings are somewhat surprising considering that literature points out that the acquisition of verbs requires more complexive attributes than nouns (Nomikou et al., 2019). While nouns can be drawn from relatively established referential frames, verbs refer to events that are complex and less transparent to single out concrete semantic features (e.g., Gentner, 1982; Hirsh-Pasek and Golinkoff, 2006; Heller and Rohlfing, 2017). In this vein, other studies suggest the possibility that the acquisition of verbs benefits from multimodal presentations comprising additional semantic features (Goodrich and Hudson Kam, 2009; Kita et al., 2017; Wakefield et al., 2018b). For example, Mumford and Kita (2014) argue that extracting relevant features is one of the key elements in verb learning. With this in mind, it would be reasonable to expected that verbs were better learned due to the broader variety of semantic features within the PRG condition. While the omnibus 3-way ANOVA does not confirm significant effects between the word classes, a descriptive level of analysis shows that the studied children learned verbs accompanied with PRG as well as they learned nouns accompanied by PRG. In contrast, the children learned only half as many verbs as nouns when both word classes were accompanied with FEG. This descriptive analysis indicates the possibility that with increased power, various forms of gestures might be a method that responds better to demands in verb acquisition. Further research is needed to investigate whether PRG are particularly conducive to the acquisition of verbs.

Our second premise outlined in the introduction is that the movements themselves also play a role in learning with PRG. We have argued that children’s production of novel words becomes enhanced with PRG because children can focus more on the label provided. While we found enhanced word learning effects in the long term, we did not investigate how different gesture conditions influenced children’s attention. Future research can thus follow up an investigate how different iconic gesture versions affect children’s attention.

## Limitations

As mentioned above, our study has some limitations. First, we have argued that reduction in gestures can enrich children’s semantic word knowledge by enabling a deeper encoding process induced by the reduced movement processing. It remains an open question whether the use of different iconic gestures would result in a similar learning effect.

Another limitation is the fact that children performed poorly in the production task, whereas they reached high scores in word reception. It seems reasonable to assume that the children’s production scores would have been higher if the target words had been presented more frequently. However, the PRG condition required us to reduce each gesture only twice, to ensure the reductions between the different versions were noticeable. Consequently, the occurrence of each target word was limited to three times.

Finally, we decided to desynchronize the presentation of the spoken word from its accompanying iconic gesture. This was necessary to ensure that the presentation of the word was the same in each repetition. Normally, words are produced simultaneously with gestures. Consequently, as a gesture is reduced, the accompanying word’s phonological form is also reduced. Since this confounds the effects of word with gesture presentation, we attempted to design our study so that it would avoid this problem. The desynchronization of the gesture and the word might have had an effect on children’s learning outcome, as it seems easier for children to pay sufficient attention to a gesture and the target word. One way to perform variations of gestures simultaneously with the target word would be to use a social robot as storyteller. Despite a small sample size, this concept has shown promise in positively influencing word learning with PRG in preschool children (Mertens, 2017).

## Summary and Conclusion

With our study, we have demonstrated that children’s long-term word learning becomes enhanced through exposure to progressively reduced iconic gestures (PRG). The novelty of our research resides in the systematic description and experimental investigations of gestures that vary in their form when repeated during a word learning scenario. We have demonstrated the effects of PRG on productive word learning and offered thorough explanations. Our findings contribute to the growing evidence that a key element in supporting long-term learning processes is to reduce the learning content during its visual presentation. In this sense, the condition, in which a novel word was accompanied by PRG experienced reduction and thus a progressive abstraction of semantic features related to it. Our study also contributes novel findings to gestural research on language learning in children since the participants were older than previously studied (Rohlfing, 2019). Regarding nonverbal behavior and learning, it remains a question for further research whether reduction affects learning in other tasks similarly, for instance, in explicit learning situations such as math.

## Data Availability Statement

The raw data supporting the conclusions of this article will be made available by the authors, without undue reservation.

## Ethics Statement

The studies involving human participants were reviewed and approved by University of Paderborn. Written informed consent to participate in this study was provided by the participants’ legal guardian/next of kin. Written informed consent was obtained from the individual(s) for the publication of any potentially identifiable images or data included in this article. Drawings: Copyright © 2013 Joy Katzmarzik leap4joy graphics; reprinted with permission.

## Author Contributions

UM and KR conceived and designed the study. UM piloted the study, recruited participants, conducted the study, and analyzed the data. UM and KR drafted the manuscript. Both authors commented on, edited, and revised the manuscript prior to submission. Both authors contributed to the article and approved the submitted version.

## Conflict of Interest

The authors declare that the research was conducted in the absence of any commercial or financial relationships that could be construed as a potential conflict of interest.

## Supplementary Material

Supplementary Figure 1 The Figure shows the gesture versions and pictures of the target words “staksen”, “retschen” and “krauchen” (Copyright © 2013 Joy Katzmarzik leap4joy graphics; reprinted with permission).

Supplementary Figure 2 The Figure shows the gesture versions and pictures of the target words “gliddern”, “Beisa” and “Ralle” (Copyright © 2013 Joy Katzmarzik leap4joy graphics; reprinted with permission).

Supplementary Figure 3 The Figure shows the gesture versions and pictures of the target words “Alk” and “Fennek” (Copyright © 2013 Joy Katzmarzik leap4joy graphics; reprinted with permission).
