# Supplementary material for: Progressive Reduction of Iconic Gestures Contributes to School-Aged Children’s Increased Word Production
Source: Front Psychol. 2021 Apr 26;12:651725. doi: 10.3389/fpsyg.2021.651725 (PMC8107226; doi:10.3389/fpsyg.2021.651725)
Supplement: Supplementary Figure 1 — The Figure shows the gesture versions and pictures of the target words “staksen”, “retschen” and “krauchen” (Copyright © 2013 Joy Katzmarzik leap4joy graphics; reprinted with permission). [file Data_Sheet_1.docx]

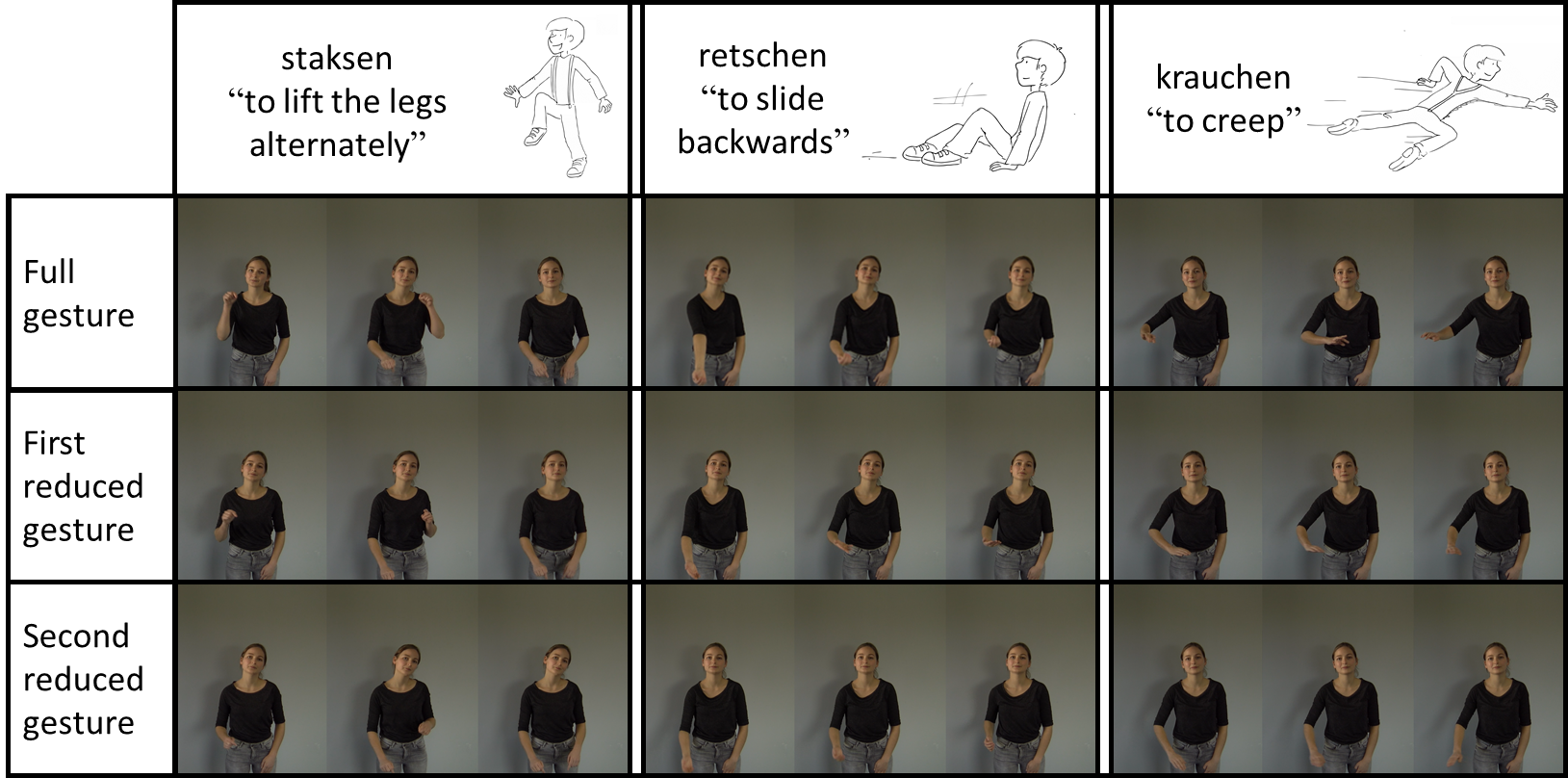


Figure 1. The Figure shows the gesture versions and pictures of the target words “staksen”, “retschen” and “krauchen” (Copyright © 2013 Joy Katzmarzik leap4joy graphics; reprinted with permission).


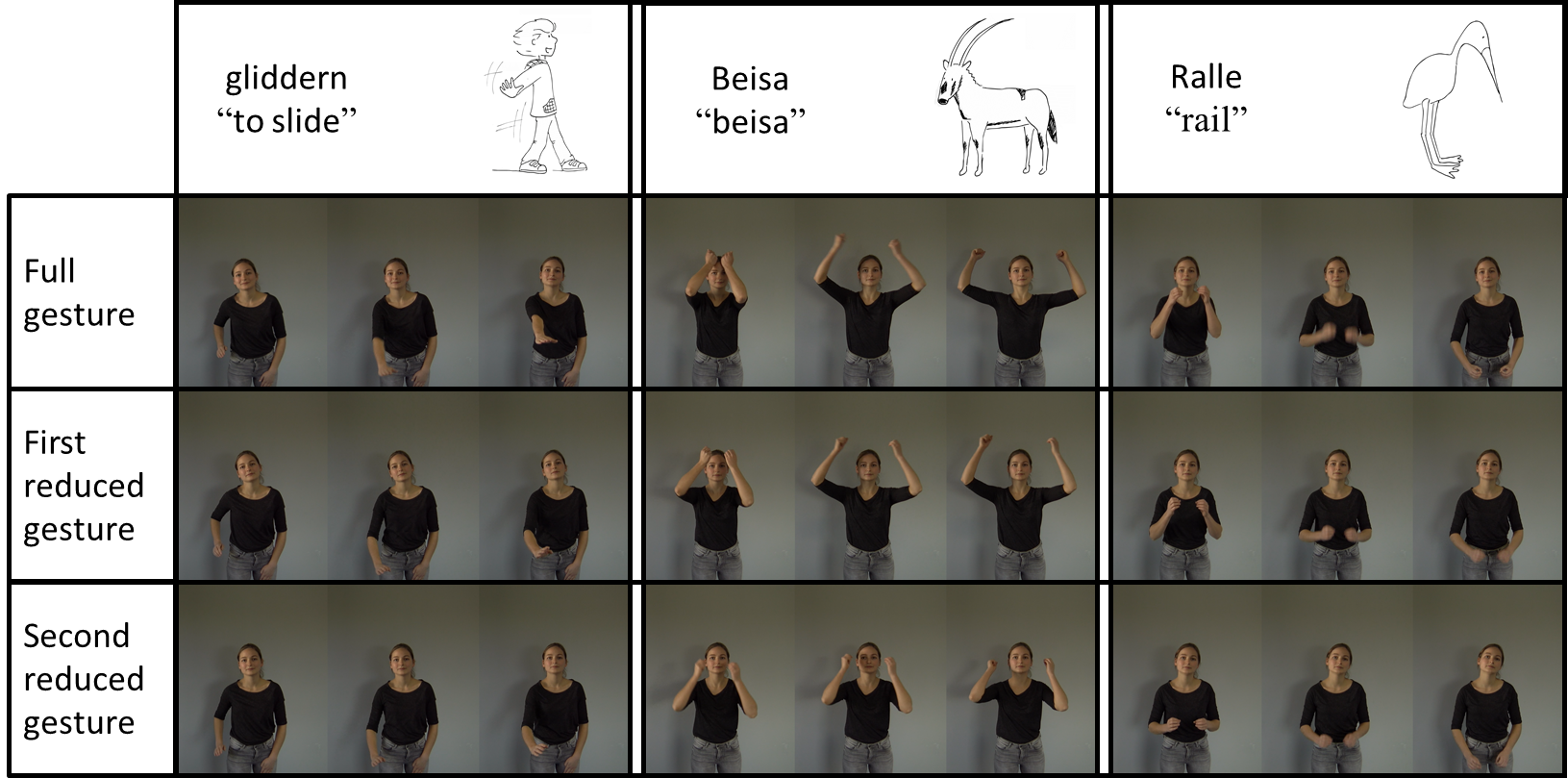


Figure 2. The Figure shows the gesture versions and pictures of the target words “gliddern”, “Beisa” and “Ralle” (Copyright © 2013 Joy Katzmarzik leap4joy graphics; reprinted with permission).


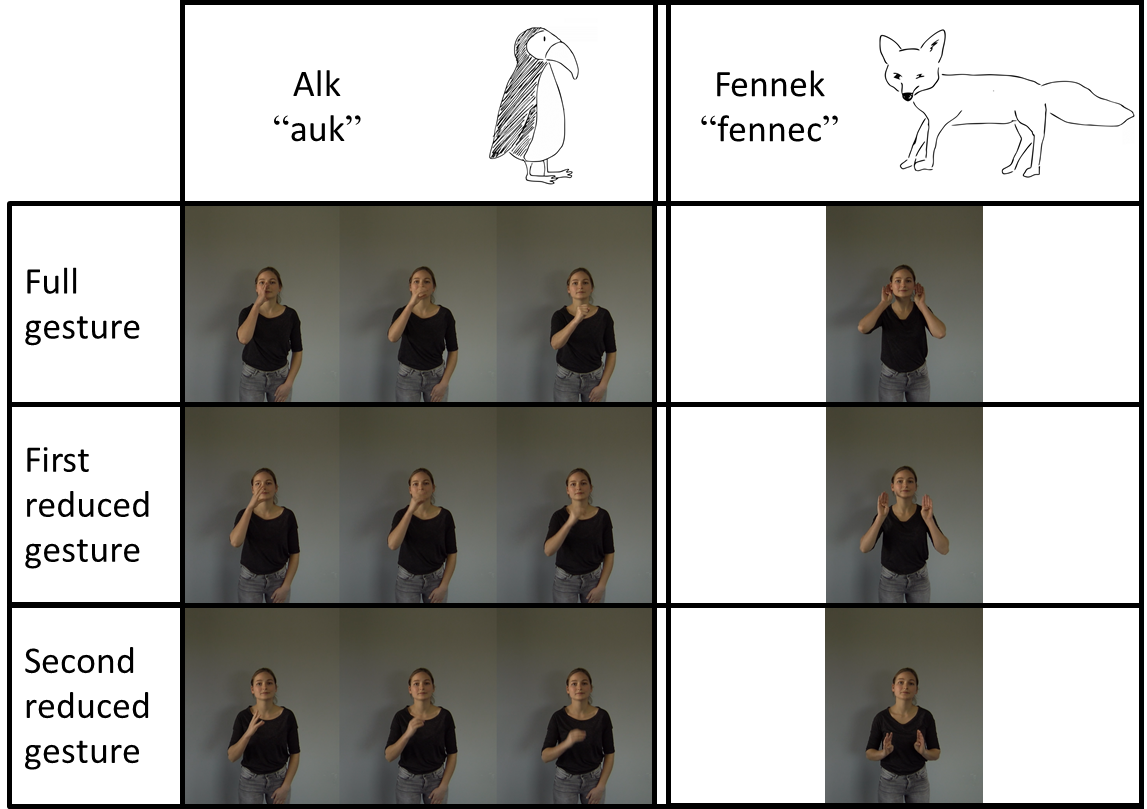


Figure 3. The Figure shows the gesture versions and pictures of the target words “Alk” and “Fennek” (Copyright © 2013 Joy Katzmarzik leap4joy graphics; reprinted with permission).
